# A Necroptosis-Related Gene Signature to Predict the Prognosis of Skin Cutaneous Melanoma

**DOI:** 10.1155/2022/8232024

**Published:** 2022-11-16

**Authors:** Yihui Xie, Ziqian Xu, Xingyu Mei, Weimin Shi

**Affiliations:** Department of Dermatology, Shanghai General Hospital, Shanghai Jiao Tong University School of Medicine, Shanghai, China

## Abstract

The prognosis of skin cutaneous melanoma (SKCM) remains poor, and patients with SKCM show a poor response to immunotherapy. Thus, we aimed to identify necroptosis-related biomarkers, which can help predict the prognosis of SKCM and improve the effectiveness of precision medicine. Data of SKCM were obtained from The Cancer Genome Atlas (TCGA) and GEO databases. TCGA samples were classified into two clusters by consensus clustering of necroptosis-related genes. Univariate Cox and least absolute shrinkage and selection operator regression analyses led to the identification of 11 genes, which were used to construct a prognostic model. GSE65904 was used as the test set. Principal component, t-distributed stochastic neighbor embedding, and Kaplan–Meier survival analyses indicated that samples in the train and test sets could be divided into two groups, with the high-risk group showing a worse prognosis. Univariate and multivariate Cox regression analyses were performed, and a nomogram, calibration curve, and time-dependent receiver operating characteristic curve were constructed to verify the efficacy of our model. The 1-, 3-, and 5-year areas under the receiver operating characteristic curves for the train set were 0.702, 0.663, and 0.701 and for the test set were 0.613, 0.627, and 0.637, respectively. Moreover, we performed Gene Ontology and Kyoto Encyclopedia of Genes and Genomes pathway enrichment analyses between the high- and low-risk groups. Single sample gene set enrichment analysis, immune cell infiltration analysis, tumor microenvironment scores, immune checkpoint analysis, and half-maximal inhibitory concentration prediction indicated that the high-risk group showed weaker antitumor immunity; further, the response to immune checkpoint inhibitors was worse, and the high-risk group was sensitive to fewer antitumor drugs. Tumor mutational burden analysis, Kaplan–Meier survival analysis, and correlation analysis between risk score and RNA stemness score revealed that the high-risk group with low tumor mutational burden and high RNA stemness score was potentially associated with poor prognosis. To conclude, our model, which was based on 11 necroptosis-related genes, could predict the prognosis of SKCM; in addition, it has guiding significance for the selection of clinical treatment and provides new research directions to enhance necroptosis against SKCM.

## 1. Introduction

Skin cutaneous melanoma (SKCM) is the most life-threatening skin cancer [1–5]. Its incidence continues to increase across the world, particularly in western countries [6]. The occurrence of SKCM is related to the environment and diverse intrinsic factors. UV radiation is one of the main extrinsic risk factors of SKCM [7]. Under the influence of various factors, melanin switches its role from antioxidant to prooxidant, and the level of intracellular oxygen free radicals increases, resulting in damage to DNA molecules; overactivation of many cell signaling pathways; and uncontrolled proliferation, dedifferentiation, and immortality of specific cells, eventually leading to cancer [8, 9]. Among the intrinsic factors, the number of melanocytic nevi, genetic susceptibility, and family history play a key role in SKCM occurrence [4].

At present, the treatment strategy of melanoma has changed greatly: surgical resection is still the most important treatment for patients with early melanoma, while immune regulation focuses on the activation of tumor-infiltrating lymphocytes in patients with advanced or metastatic melanoma. Therapies are gradually taking shape [10, 11]. In a variety of cancers, miRNAs and circRNAs have been found to be associated with tumor resistance and chemoradiotherapy sensitivity [12]. In uveal melanoma, miR-125b, miR-20a, miR-146a, miR-181a, miR-155, miR-223, and other miRNAs have been found to be in disorder [13]. lncRNAs were significantly associated with clinicopathological features [14]. Although drugs targeting miRNA or circRNA have not been widely used, various monoclonal antibody drugs targeting PD-1 and CTLA-4 are gradually used in clinical practice. Although monoclonal antibody therapy has significantly improved patient outcomes, not all patients respond to treatment with immune checkpoint inhibitors [15]. Therefore, more effective stratification of tumor patients with the help of bioinformatics will help us to screen patients who may respond to immunotherapy.

Bioinformatics analysis is a fundamental pillar of the precision treatment of diseases, including tumors. Bioinformatics analysis processes analyze massive sample data through algorithms, so as to effectively analyze the complex mechanisms (including genetic and epigenetic regulation) behind diseases. Candido et al. revealed the relationship between epigenetic regulation of IL6 signaling in tumors [16]. In nonneoplastic diseases, researchers have used bioinformatics to identify key genetic and epigenetic changes associated with pesticide exposure [17]. In melanoma, new markers are continuously exposed through bioinformatics technologies, and multiple markers including IL27, CXCL8, THBS1, and KIT have been identified to be associated with melanoma metastasis and treatment outcomes [18, 19]. Bioinformatics analysis plays an increasingly important role in the exploration of new disease diagnosis and treatment and prognostic markers.

Apoptosis resistance poses a major threat to the success of cancer treatment [20]. Circumventing the apoptotic pathway to induce cell death is an effective solution. Adjuvant high-dose interferon and ipilimumab are associated with survival benefits, but they are highly toxic. In comparison with traditional therapy, programmed cell death protein-1 inhibitor and BRAF/MEK-targeted therapies were recently reported to significantly improve patient survival, but several patients were found to develop primary or acquired resistance after initial response [21]. Necroptosis, a programmed cell death mechanism, is an efficient way to induce cell death and is crucial to patient prognosis [22]. TNF-*α* and its receptors promote the assembly of the receptor-interacting protein kinase 1- (RIPK1-) RIPK3-mixed lineage kinase-like (MLKL) signaling complex, and RIPK3-mediated phosphorylation of MLKL leads to its translocation to the plasma membrane to initiate membrane damage, which induces necroptosis [23]. Although necroptosis shares some similarities with apoptosis, it exhibits morphological features similar to those of necrosis [24]. Necroptosis resistance in SKCM is evidently associated with the loss of RIPK3 [25]. P65/RelA and NF-*κ*B fragments generated by active RIPK3 regulate tumorigenicity, cellular metabolism, and stemness characteristics [26]. Necroptosis, defined by the release of inflammatory mediators, alters the inflammatory state of the tumor microenvironment (TME) and influences the response to drug sensitivity in cancer [27]. A previous study reported that the topoisomerase inhibitor SN38 induces colon adenocarcinoma cell death by enhancing necroptosis [28]. Furthermore, bufalin has been found to inhibit human breast cancer tumorigenesis through necroptosis [29].

This study was conducted using data obtained from The Cancer Genome Atlas (TCGA) and the GEO databases. We constructed a prediction model comprising 11 necroptosis-related genes, and the efficiency of this model as an independent predictor was verified by time-dependent receiver operating characteristic (ROC) analysis. The single sample gene set enrichment analysis (ssGSEA), immune cell infiltration, immune checkpoint analysis, and prediction of the half-maximal inhibitory concentration (IC50) have guiding significance for immunotherapy. The high-risk group showed a high RNA stemness score (RNAss) implying the ability to progress, metastasize, and drug resistance. We believe that our findings will guide treatment selection and provide a new research direction to enhance necroptosis against SKCM.

## 2. Materials and Methods

### 2.1. Data Collection

RNA sequencing data and relevant clinical information of SKCM were downloaded from TCGA (https://portal.gdc.cancer.gov/). To reduce statistical bias, we excluded SKCM samples with missing and/or short (<30 days) overall survival (OS) values, which led to the identification of 447 samples.

The test set GSE65904 was obtained from the GEO database; on excluding SKCM samples with missing and/or short (<30 days) OS values, we attained 207 samples.

**Figure 1:**
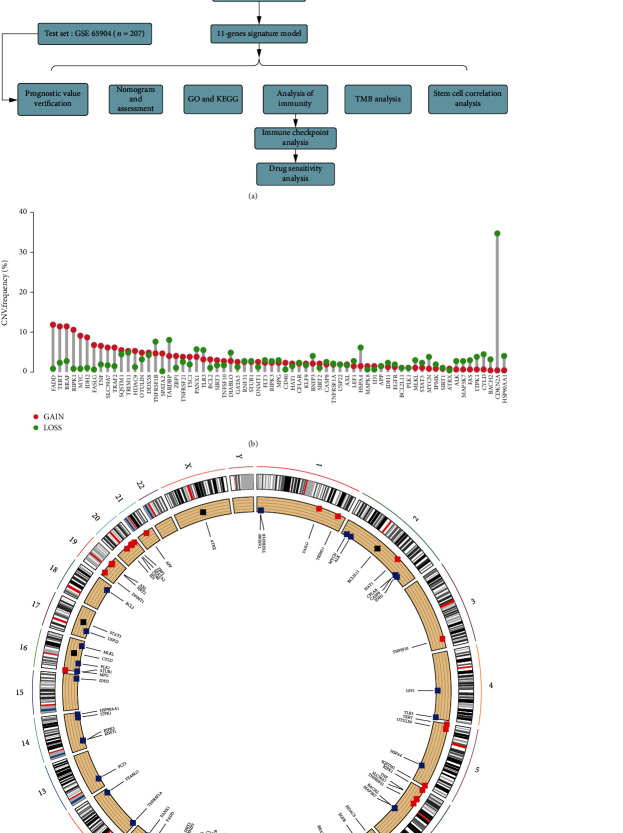
Global changes in necroptosis-related genes. (a) Flow chart. (b) CNV frequency of necroptosis-related genes. (c) The location of CNVs of necroptosis-related genes on chromosomes.

Figure 1(a) describes which samples were used at each stage of statistical analyses. The “maftools” package was applied to analyze copy number variations.

### 2.2. Selection of Necroptosis-Related Genes

The necroptosis gene set M24779.gmt, which contains eight genes, was downloaded from the Gene Set Enrichment Analysis website (http://www.gsea-msigdb.org/gsea/index.jsp). By searching necroptosis-related literature, we identified a total of 67 necroptosis-related genes (Appendix T1) [20, 30].

### 2.3. Consensus Clustering

Consensus clustering was used to identify distinct necroptosis-related patterns relating to the expression of necroptosis-related genes. The number of clusters and their stability were determined by a consensus clustering algorithm, which was executed using the “ConsensuClusterPlus” package.

### 2.4. Establishment and Validation of the Risk Signature

Based on the clinical data for SKCM samples in TCGA, univariate Cox (uni-Cox), fold cross-validation, and least absolute shrinkage and selection operator (LASSO) regression analyses were conducted to choose model genes (*p* < 0.001). We performed 1,000 times repetitions to guarantee the stability of our classification. Principal component analysis (PCA), t-distributed stochastic neighbor embedding (t-SNE), and Kaplan–Meier survival analyses were performed using the “Rtsne” R package.

### 2.5. Nomogram and Calibration

uni-Cox and multivariate Cox (multi-Cox) regression analyses were applied to assess whether risk score and clinical characteristics were independent variable factors. A ROC curve was constructed to compare varied factors in predicting outcome. Finally, with the “rms” R package, risk score, age, and tumor stage were used to construct a nomogram to predict 1-, 3-, and 5-year OS, and correction curves were generated based on the Hosmer–Lemeshow test.

### 2.6. Gene Ontology (GO) and Kyoto Encyclopedia of Genes and Genomes (KEGG) Pathway Enrichment Analyses

We used the “limma” package to identify differentially expressed genes (DEGs) (*q* < 0.05 and |logFC| > 1), and subsequently, they were subjected to GO and KEGG pathway enrichment analyses with the R package “clusterProfiler.”

### 2.7. Immune-Related Analysis

ssGSEA was performed with the “GSVA” R package. The “CIBERSORT” R package was used to analyze the correlation in immune cell infiltration among 11 necroptosis-related genes. The “ggpubr” R package was applied to assess TME scores and immune checkpoint activation between the low- and high-risk groups, and “maftools” was used to analyze tumor mutational burden (TMB) of necroptosis-related genes in these groups.

### 2.8. Exploration of the Model in Clinical Treatment

To determine the therapy response of patients with SKCM, we used the R package “pRRophetic” to evaluate IC50, as per the Genomics of Drug Sensitivity in Cancer database (GDSC) (https://www.cancerrxgene.org/).

### 2.9. Correlation Analysis of Risk Score with RNA Stemness Score

A correlation analysis between risk score and RNAss was performed by Spearman's method using the “cor. Test” command and the R package “limma,” followed by visualization with the R package “corrplot.”

## 3. Results

### 3.1. Necroptosis-Related Genes in Patients with SKCM

Figure 1(a) shows the research flow. First, we explored the mutational status of 67 necroptosis-related genes (i.e., regulators) in TCGA samples. CDKN2A deletions were found to be the most prevalent (Figure 1(b)). Alterations in these regulators characterized by copy number variations on the chromosome were identified (Figure 1(c)).

### 3.2. Identification of Subtypes and Their Distinct Necroptosis Patterns

**Figure 2:**
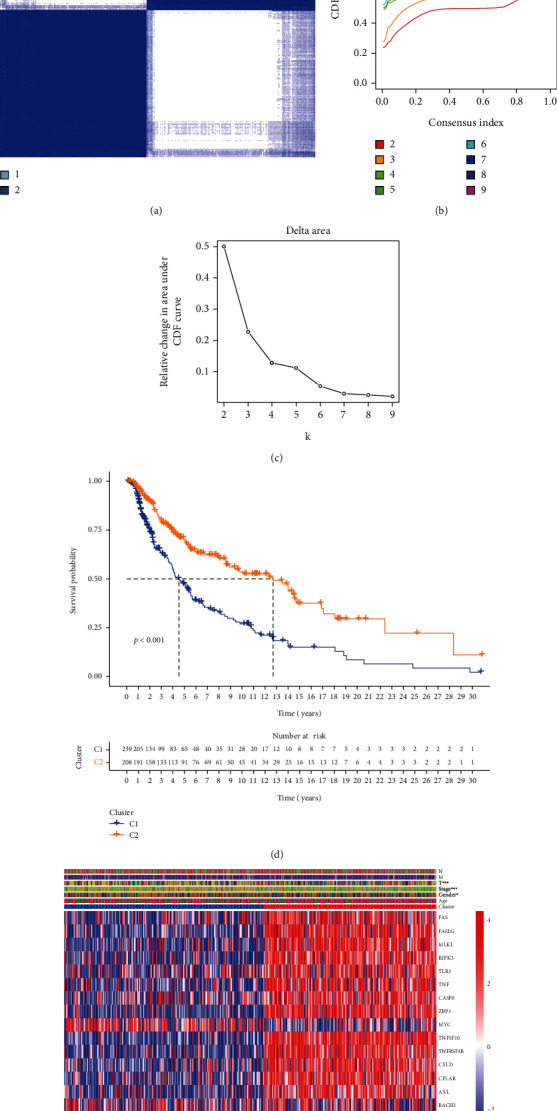
Consensus clustering of SKCM samples among necroptosis-related genes. (a) The consensus score matrix of all samples when *k* = 2. (b) In the CDF graph when *K* takes different values, when *k* = 2, the CDF decline gradient is the smallest, and the CDF reaches an approximate maximum value, and the cluster analysis results at this time are the most reliable. (c) The area under CDF change shows the relative change in the area under the CDF curve compared to *K* and *K* − 1. (d) Survival analysis of samples in clusters 1 and 2 in TCGA. (e) Heatmap of necroptosis-related genes in different clusters.

SKCM samples were divided into two clusters (cluster 1 and cluster 2) by consensus clustering. In cancer research, consensus clustering classifies groups with common biological characteristics potentially existing but unknown inner. When *k* = 2, the classification is the clearest on the consensus matrix heatmap, the cumulative distribution function (CDF) reaches an approximate maximum, and the growth rate of the area under the CDF curve is close to 0, indicating that clusters have the highest concordance at this point (Figures 2(a)–2(c) and Supplementary Figure S1A). Herein, the survival curves showed significant differences between cluster 1 and 2, and the survival advantage of cluster 2 was higher than that of cluster 1 (Figure 2(d)). The heatmap depicted the expression of necroptosis-related genes between these clusters (Figure 2(e)).

### 3.3. Model Construction and Verification

**Figure 3:**
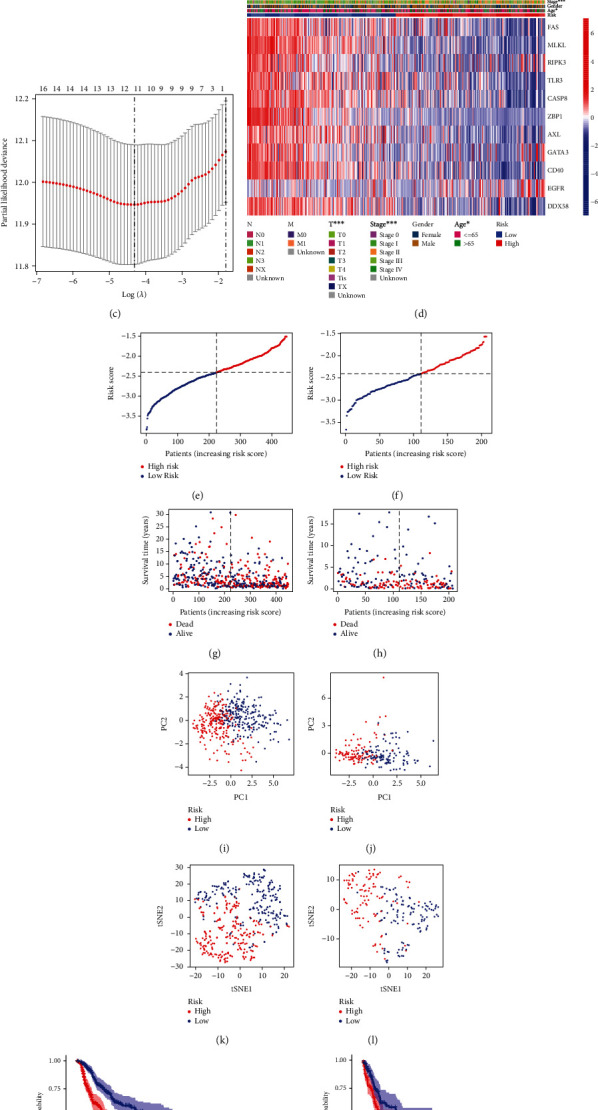
Selection of necroptosis genes associated with prognosis. (a) Univariate Cox analysis of the candidate necroptosis-related gene set. (b) Cross-validation for variable selection in the LASSO model. (c) The LASSO coefficient spectrum of 11 necroptosis-related genes. (d) The expression profiles of 11 prognostic genes. (e, f) Exhibition of necroptosis-related gene model based on risk score of the train and test, respectively. (g, h) Survival time and survival status between low- and high-risk groups in the train and test, respectively. (i, j) The PCA between low- and high-risk groups in the train and test, respectively. (k, l) The t-SNE between low- and high-risk groups in the train and test, respectively. (m, n) Kaplan–Meier survival curves of OS of patients between low- and high-risk groups in the train and test, respectively.

Through uni-Cox regression analysis, 13 necroptosis-related genes were found to be significantly associated with OS (*p* < 0.001), playing a protective role (Figure 3(a)). To avoid overfitting the prognostic signature, we performed a LASSO regression analysis on these genes; consequently, we extracted 11 genes when the first-rank value of Log(*λ*) was the minimum likelihood of deviance (Figures 3(b) and 3(c)).

Risk score was calculated using this formula: [FAS × (−0.102814625730389) + MLKL × (−0.0186470527508156) + RIPK3 × 0.117717056112214 + TLR3 × (−0.227081489914472) + CASP8 × (−0.131637762064241) + ZBP1 × (−0.279134792483509) + AXL × (−0.139002477382656) + GATA3 × 0.0483383170281774 + CD40 × (−0.109447476720426) + EGFR × 0.225336554991422 + DDX58 × (−0.00389392183935227)].

Heatmap depicted the distribution of the 11 genes in the high- and low-risk groups (Figure 3(d)).

Using the aforementioned formula and based on the median, the TCGA dataset, i.e., the training set, and GSE65904, i.e., the test set, were classified into low- and high-risk groups. Moreover, using the risk score formula, we compared the distribution of risk score, survival status, survival time, PCA, and t-SNE analysis data between the high- and low-risk groups in the training and test sets (Figures 3(e)–3(l)). A clear distributional difference was found between the high- and low-risk groups. The high-risk group showed worse prognoses in both the training and test sets (Figures 3(m) and 3(n)).

### 3.4. Nomogram Construction

**Figure 4:**
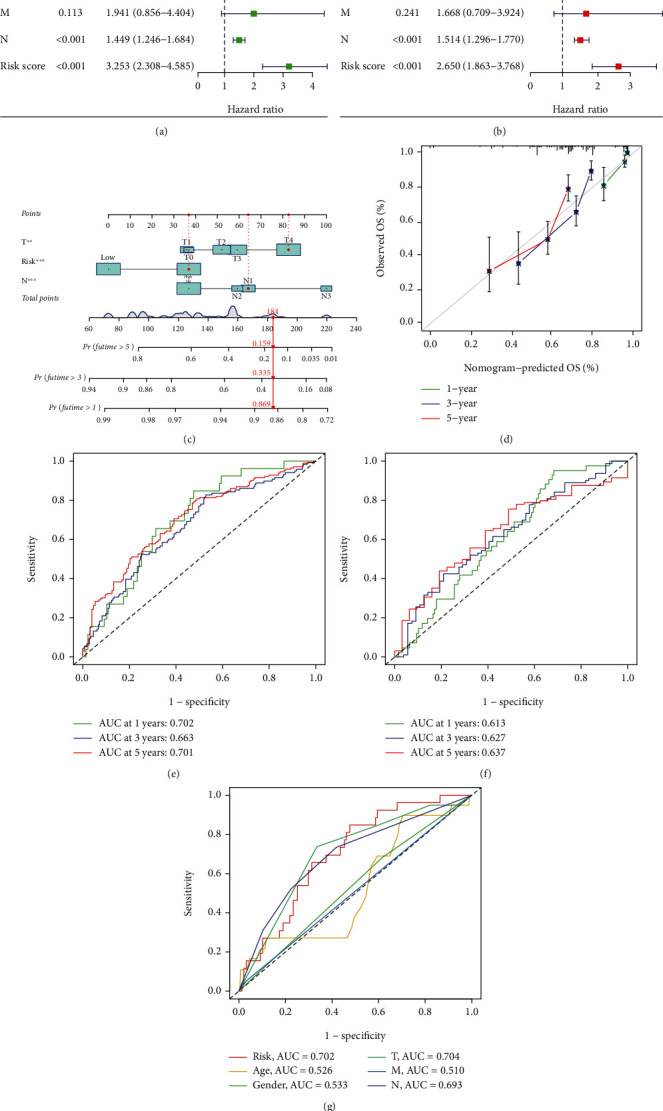
Nomogram and assessment of the risk model. (a) uni-Cox analyses of clinical factors and risk score with OS. (b) multi-Cox analyses of clinical factors and risk score with OS. (c) The nomogram that integrated the risk score and tumor stage predicted the probability of the 1-, 3-, and 5-year OS. (d) The calibration curves for 1-, 3-, and 5-year OS. (e, f) The 1-, 3-, and 5-year ROC curves of the train and test, respectively. (g) The 1-year ROC curves of risk score and clinical characteristics.

The hazard ratio of risk score and 95% confidence interval were 3.253 and 2.308–4.585 (*p* < 0.001), respectively, in uni-Cox regression and 2.650 and 1.863−3.768 (*p* < 0.001), respectively, in multi-Cox regression (Figures 4(a) and 4(b)). In addition, we found two independent prognostic factors: T stage (1.350, 1.159−1.572, *p* < 0.001) and N stage (1.514, 1.296−1.770, *p* < 0.001) (Figure 4(b)).

Based on the three independent prognostic factors, namely, risk score, T stage, and N stage (all *p* < 0.001 in multi-Cox regression), we constructed a nomogram to predict 1-, 3-, and 5-year OS incidence among patients with SKCM (Figure 4(c)). We also plotted a calibration curve to demonstrate that the nomogram was in good agreement with the 1-, 3-, and 5-year OS predictions (Figure 4(d)).

### 3.5. Risk Model Assessment

ROC analysis was performed to assess the sensitivity and specificity of the model for prognosis, with the area under the ROC curve (AUC) serving as the outcome. The 1-, 3-, and 5-year AUCs for the training set were 0.702, 0.663, and 0.701, respectively, and the 1-, 3-, and 5-year AUCs for the test set were 0.613, 0.627, and 0.637, respectively, indicating that our prognostic model had good predictive performance (Figures 4(e) and 4(f)). In the 1-year ROC of the training set, in comparison with the five clinically independent prognostic factors, the model was found to be more predictive (Figure 4(g)).

### 3.6. Enrichment Analysis

**Figure 5:**
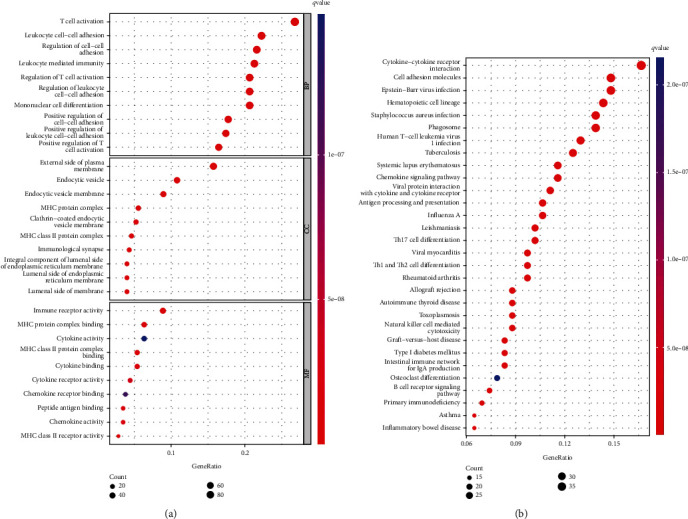
GO and KEGG enrichment analyses. (a) Bubble plot of GO enrichment analysis of differential genes between high- and low-risk groups. (b) Bubble plot of KEGG enrichment analysis of differential genes between high- and low-risk groups.

To assess differences in biological functions between the different risk groups, DEGs were subjected to GO and KEGG pathway enrichment analyses (Figures 5(a) and 5(b) and Supplementary Figure S1B). The most abundant biological processes included T cell activation, leukocyte cell adhesion, regulation of cell-cell adhesion, leukocyte-mediated immunity, monocyte differentiation, and regulation, among others. In terms of molecular functions, DEGs were primarily enriched in immune receptor activity, MHC protein complex binding, MHC class II protein complex binding, cytokine activity, and cytokine binding, among others. KEGG pathway enrichment analysis revealed that the different risk group was highly correlated with the cytokine-cytokine receptor interaction pathway.

### 3.7. Investigation of Immunity Factors and Clinical Treatment in the Risk Groups

**Figure 6:**
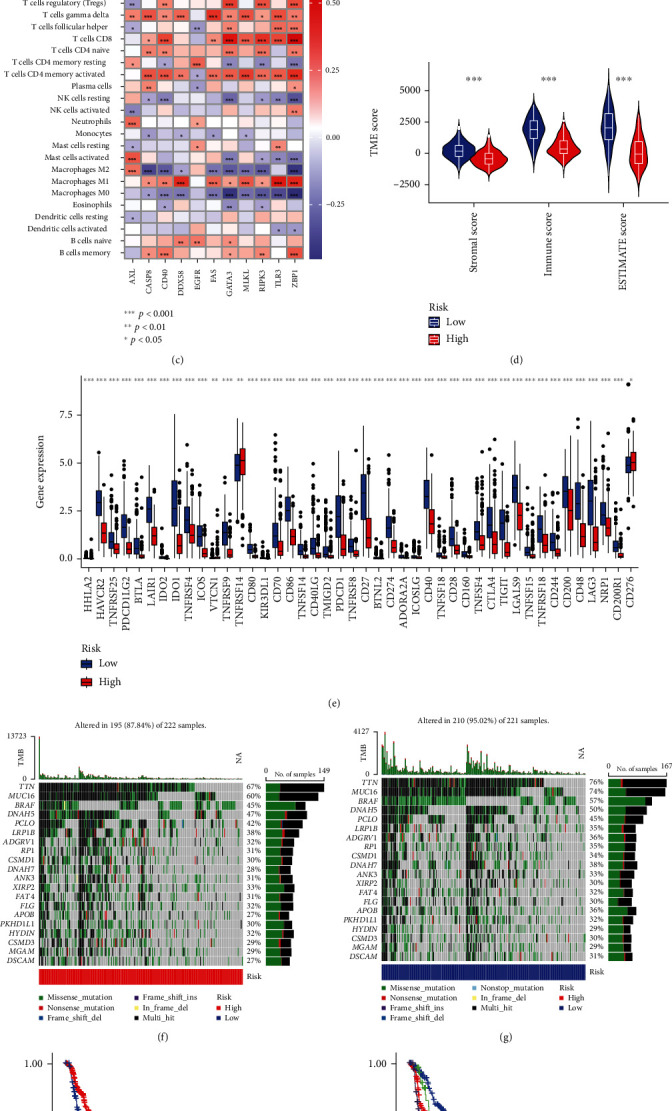
Immune-related analysis. (a) The ssGSEA scores of immune cells between high- and low-risk groups. (b) The ssGSEA scores of immune functions between high- and low-risk groups. (c) The correlation of 11 model genes with immune cells. (d) The comparison of immune-related scores between high- and low-risk groups. (e) The different checkpoint expressions between high- and low-risk groups. (f) The top 20 mutated genes in the high-risk group. (g) The top 20 mutated genes in the low-risk group. (h) Kaplan–Meier survival curves of OS of patients between low- and high-TMB groups. (i) Kaplan–Meier survival curves of OS of patients between high- and low-risk groups combined with high- and low-TMB groups.

We analyzed differences in immune cells and immune function between the high- and low-risk groups (Figures 6(a) and 6(b)). In comparison with the high-risk group, the low-risk group showed higher scores of various immune cells and immune functions. The correlation analysis between immune cells and the 11 genes in the model revealed that M1 macrophages, memory B cells, plasma cells, T cells CD4 memory activated, T cells CD8, and T cells gamma delta were negatively correlated with the 11 genes, whereas M0 macrophages, M2 macrophages, monocytes, and NK cells resting were positively correlated with the 11 genes (Figure 6(c) and Supplementary Figure S2).

The high-risk group showed lower stromal, immune, and estimate scores and exhibited a different TME than the low-risk group (Figure 6(d)). Further, most immune checkpoints in the low-risk group were highly expressed, while in the high-risk group, highly expressed immune checkpoints (such as TNFRSF14 and CD276) were rare, indicating that targeted therapy might not be effective in the high-risk group (Figure 6(e)). Immune checkpoint inhibitors can be chosen depending on different risk groups. Drug sensitivity analysis revealed that the high-risk group showed a lower IC50 for 25 cancer treatment drugs (Supplementary Figure S3A).

Patients with higher TMB showed an enhanced response, long-term survival, and durable clinical benefit when treated with immunotherapy [31]. Herein, we found that the low-risk group had higher TMB, demonstrating better immunotherapy response, and higher survival advantage (Figures 6(f)–6(i) and Supplementary Figure S3B).

### 3.8. Cancer Stem Cell Correlation Analysis

**Figure 7:**
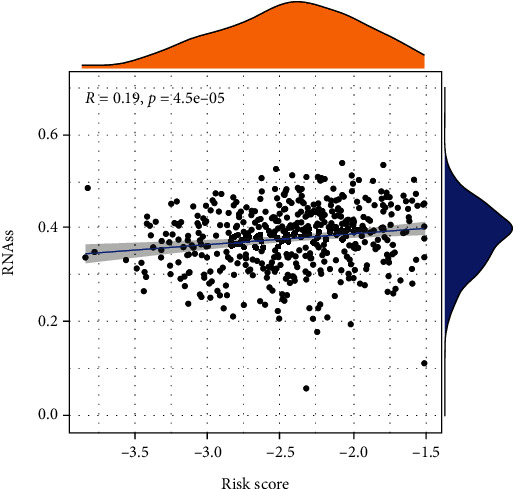
The relationship between risk score and tumor cell stemness.

The risk score is proportional to the stemness of tumor cells. Patients with a high-risk score showed relatively stronger stemness characteristics of tumor cells and poor prognosis (Figure 7).

## 4. Discussion

The high mortality rate of SKCM has driven several advancements in treatment techniques. In comparison with traditional methods such as chemotherapy and radiotherapy, immunotherapy is a new treatment method for patients with SKCM; however, its response is not the same. SKCM stimulates an immunogenic response, and the production of specific cytotoxic T lymphocytes (CTLs) is induced, which kill SKCM cells via the Fas/FasL-independent and particle-dependent lytic pathway; however, SKCM cells often evade immune destruction [32].

Herein, the established prognostic model comprised 11 necroptosis-related genes (FAS, MLKL, RIPK3, TLR3, CASP8, ZBP1, AXL, GATA3, CD40, EGFR, and DDX58). The risk score could predict immune status, response to immunotherapy, and prognosis. In clinical practice, risk scores can help individualize treatment for patients, select appropriate drugs, and improve the success rate and efficiency of treatment.

Necroptosis is a regulated, caspase-independent, immunogenic mode of cell death and is primarily mediated by RIP1, RIP3, and MLKL. It is evidently induced by Toll-like receptors (TLRs), tumor necrosis factor receptors, interferons, and intracellular RNA and DNA sensors [20, 33, 34]. RIP1 defines cell survival or death; it recruits and activates RIPK3, which interacts with RIPK1 to form necrosomes. RIPK3 then phosphorylates MLKL, which in turn oligomerizes and translocates to the plasma membrane, leading to membrane permeabilization and necroptosis; for this reason, MLKL modification is critical [34, 35]. Caspase-8 cleaves RIPK1 as well as RIPK3 and activates apoptosis [20]. Necrosomes, composed of RIP1, RIP3, and Fas-related death domain proteins, activate pseudokinase-mixed lineage kinases. Necroptosis has been reported to play a vital role in tumorigenesis, antitumor immunity activation, and cancer therapy. Targeting necrosomes has been suggested to cause immunogenic reprogramming in the TME [36]. A cytoplasmic death-inducing signaling complex, comprising RIPK1, TRADD, caspase-8, and FADD (FAS-associated death domain protein), called complex II, induces caspase-8 activation and participates in necroptosis pathway activation. RIPK1 mediates signaling downstream of tumor necrosis factor receptor 1, TLR3, TLR4, retinoic acid-inducible gene 1, melanoma differentiation-associated protein 5, and Z-binding protein 1 (ZBP1). FAS regulates ligand-induced apoptosis, and downregulation of its expression results in resistance to FasL-mediated cell death [37, 38]. TAM (Tyro3, Axl, and Mer) kinases phosphorylate MLKL to promote necroptosis and mediate MLKL oligomerization to promote cleavage pore formation [39]. The innate immune sensor ZBP1 and the essential cell survival kinase TAK1 regulate the assembly and function of the RIPK1/RIPK3-FADD-caspase-8 cell death complex. GATA3 participates in driving tumor growth and metastasis and is thus closely associated with SKCM survival [40, 41]. EGFR regulates epithelial tissue development and homeostasis and drives tumorigenesis, and it has been recognized as a biomarker of tumor resistance (Sigismund et al., 2018).

SKCM cells evade the immune system in many ways. One such major approach is immunotherapy that activates antitumor T cells. The extent of T cell infiltration into tumors depends on innate immune activation and Batf3-dependent CD103+ dendritic cell recruitment in the TME. The relative lack of CD8+ T cells results in a poor response to immune checkpoint inhibitors. The intratumoral delivery of mRNA encoding MLKL arouses T cell antitumor response [35]. Therefore, MLKL–mRNA therapy appears promising for patients with a higher risk score.

In this study, based on risk scores, we classified all samples into high- and low-risk groups. The differences in enrichment analyses between these groups were primarily reflected in terms of T cell activation, leukocyte cell-cell adhesion, regulation of cell-cell adhesion, and leukocyte-mediated immunity, among others. SKCM induces melanoma cell death via the perforin-granzyme and Fas-Fas ligand pathways, but tumor cells are often able to evade this immune destruction [32, 42]. Cell adhesion molecules reportedly influence SKCM progression. Embryonic antigen-related cell adhesion molecule 1 (CEACAM1), a widely expressed cell-cell adhesion protein, mediates direct interactions between tumors and immune cells [43]. CEACAM1-3S is associated with enhanced immunogenicity and contributes to improved OS of patients with advanced melanoma; in contrast, CEACAM1-4L promotes tumor progression by downregulating the cell surface expression of the NKG2D ligands MICA and ULBP [44].

A more potent and durable antitumor immune response requires not only CD8+ cytotoxic T lymphocytes but also CD4+ T helper cells [45]. CD8+ CTLs induce tumor cell lysis by recognizing tumor MHC class I molecules. CD40 is a costimulatory receptor molecule involved in humoral and cellular immunity regulation [46]. Differences between the high- and low-risk groups also focused on MHC (MHC class II) protein complex binding. Most melanoma cells do not express MHC class II molecules, the main function of which is to present processed antigens, mainly derived from foreign sources, to CD4+ T lymphocytes; they are therefore essential for the initiation of antigen-specific immune responses [47]. MHC expression can guide the choice of immunotherapy for patients with SKCM [48]. Mutations identified by exome sequencing can serve as vaccine targets solely through bioinformatics prioritization based on their expression levels and MHC class II-binding capacity for rapid production as synthetic poly-neo-epitope mRNA vaccines [49]. We believe that for patients with a high-risk score, inactive necroptosis, and insufficient CD8+ and CD4+ T cell effects, a comprehensive treatment plan should ideally include the following: surgical resection with immune checkpoint inhibitor therapy and mRNA vaccines for MLKL as well as MHC class II.

Although we used many methods to evaluate our model, some limitations persist. The retrospective nature of this study makes it susceptible to various inherent biases. Furthermore, our study has no experimental validation and thus has a severe limitation. Our model is promising. In the future, samples can be collected by surgery for immunofluorescence staining to test the predictive ability of the model [50]. At the same time, more clinical data can be collected to further explore the significance of necroptosis-related genes in the treatment of SKCM. In conclusion, our experiment established a patient stratification model composed of 11 genes derived from necroptosis-related genes. This model can effectively distinguish the prognosis of SKCM patients and play a role in the precise treatment of SKCM patients in the future.

## Data Availability

The datasets presented in this study can be found in online repositories. The names of the repository/repositories and accession number(s) can be found in the article/Supplementary Material.

## Supplementary Materials

Supplementary Materials Figure S1: (A) consensus matrix of consensus clustering for *K* = 1,2,3,4,5,6,7,8,9 in TCGA. (B) Histogram of GO and KEGG enrichment analyses between high- and low-risk groups. Figure S2: the correlation analysis between risk score and immune cells. Figure S3: (A) 25 drugs that were sensitive to the high-risk group. (B) Mutations in 67 necroptosis-related genes in TCGA samples.
