# Supplementary figures and images for: A Necroptosis-Related Gene Signature to Predict the Prognosis of Skin Cutaneous Melanoma
Source: Dis Markers. 2022 Nov 16;2022:8232024. doi: 10.1155/2022/8232024 (PMC9683951; doi:10.1155/2022/8232024)

A

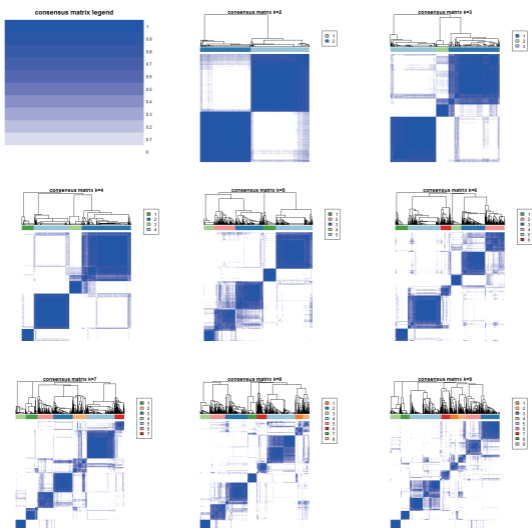

B

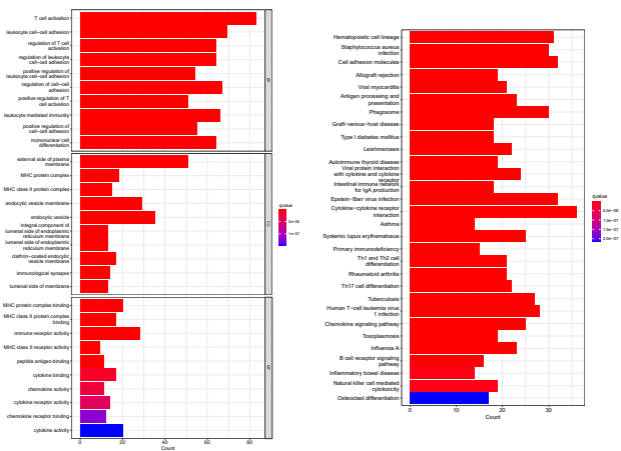

Supplement: Supplementary Materials — Figure S1: (A) consensus matrix of consensus clustering for K = 1,2,3,4,5,6,7,8,9 in TCGA. (B) Histogram of GO and KEGG enrichment analyses between high- and low-risk groups. Figure S2: the correlation analysis between risk score and immune cells. Figure S3: (A) 25 drugs that were sensitive to the high-risk group. (B) Mutations in 67 necroptosis-related genes in TCGA samples. [file 8232024.f1.zip › figure S1.pdf]

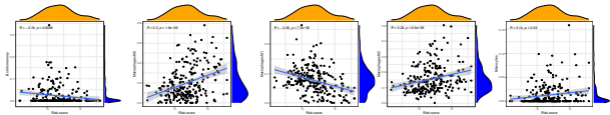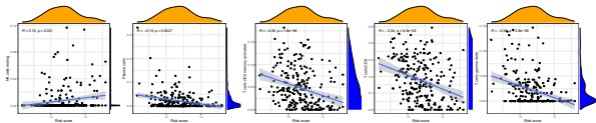

Supplement: Supplementary Materials — Figure S1: (A) consensus matrix of consensus clustering for K = 1,2,3,4,5,6,7,8,9 in TCGA. (B) Histogram of GO and KEGG enrichment analyses between high- and low-risk groups. Figure S2: the correlation analysis between risk score and immune cells. Figure S3: (A) 25 drugs that were sensitive to the high-risk group. (B) Mutations in 67 necroptosis-related genes in TCGA samples. [file 8232024.f1.zip › figure S2.pdf]

A

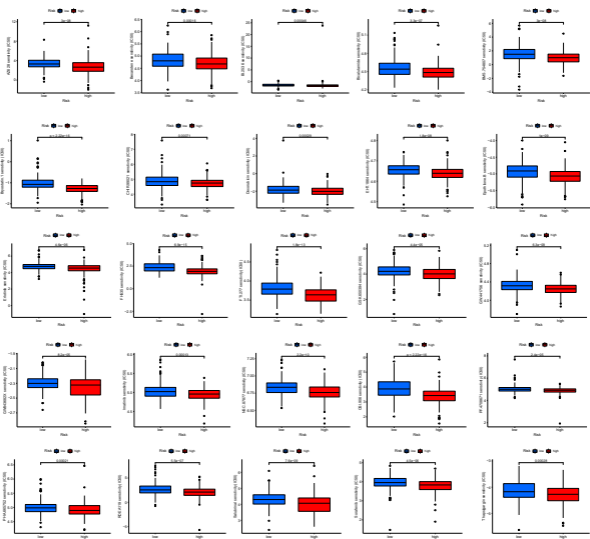

B

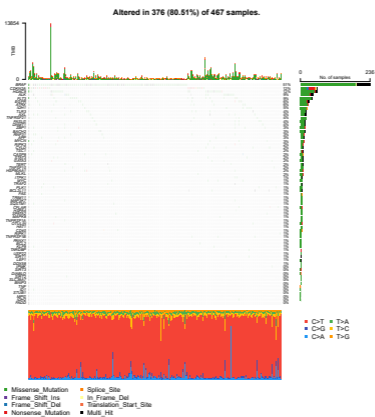

Supplement: Supplementary Materials — Figure S1: (A) consensus matrix of consensus clustering for K = 1,2,3,4,5,6,7,8,9 in TCGA. (B) Histogram of GO and KEGG enrichment analyses between high- and low-risk groups. Figure S2: the correlation analysis between risk score and immune cells. Figure S3: (A) 25 drugs that were sensitive to the high-risk group. (B) Mutations in 67 necroptosis-related genes in TCGA samples. [file 8232024.f1.zip › figure S3.pdf]
